# Serum total indoxyl sulfate levels and all-cause and cardiovascular mortality in maintenance hemodialysis patients: a prospective cohort study

**DOI:** 10.1186/s12882-022-02862-z

**Published:** 2022-06-28

**Authors:** Qian Li, Shuang Zhang, Qi-Jun Wu, Jia Xiao, Zhi-Hong Wang, Xiang-Wei Mu, Yu Zhang, Xue-Na Wang, Lian-Lian You, Sheng-Nan Wang, Jia-Ni Song, Xiu-Nan Zhao, Zhen-Zhen Wang, Xin-Yi Yan, Yu-Xin Jin, Bo-Wen Jiang, Shu-Xin Liu

**Affiliations:** 1 Department of Nephrology, Dalian Municipal Central Hospital, No.826, Xinan Road, Dalian, Liaoning 116033 P. R. China; 2 Dalian Key Laboratory of Intelligent Blood Purification, Dalian Municipal Central Hospital, Dalian, China; 3 Department of Clinical Epidemiology, Shengjing Hospital of China Medical University, Shenyang, China; 4 School of Maritime Economics and Management, Dalian Maritime University, Dalian, China

**Keywords:** Cohort study, Hemodialysis, Indoxyl sulfate, Mortality

## Abstract

**Background:**

The association between serum total indoxyl sulfate (tIS), and cardiovascular disease (CVD) and all-cause mortality is a matter of debate. In the current study we sought to determine the association, if any, between serum tIS, and all-cause and CVD-associated mortality in patients on maintenance hemodialysis (MHD).

**Methods:**

A prospective cohort study was conducted involving 500 MHD patients at Dalian Municipal Central Hospital from 31 December 2014 to 31 December 2020. Serum tIS levels were measured at baseline and classified as high (≥44.16 ng/ml) or low (< 44.16 ng/ml) according to the “X-tile” program. Besides, the associations between continuous serum tIS and outcomes were also explored. Predictors were tested for colinearity using variance inflation factor analysis. Hazard ratios (HRs) and 95% confidence intervals (CIs) were calculated using Cox proportional hazards regression models. Restricted cubic spline model was performed to assess dose-response relationships between tIS concentration and all-cause and CVD mortality.

**Results:**

During a 58-month median follow-up period, 224 deaths (132 CVD deaths) were documented. After adjustment for potential confounders, the serum tIS level was positively associated with all-cause mortality (HR = 1.02, 95% = 1.01–1.03); however, we did not detect a significant association when tIS was a dichotomous variable. Compared with the MHD population with a serum tIS level < 44.16 ng/ml, the adjusted HR for CVD mortality among those with a serum tIS level ≥ 44.16 ng/ml was 1.76 (95% = 1.10–2.82). Furthermore, we also noted the same association when the serum tIS level was a continuous variable.

**Conclusion:**

The serum tIS level was associated with higher risk of all-cause and CVD mortality among MHD patients. Further prospective large-scale studies are required to confirm this finding.

## Introduction

Chronic kidney disease (CKD) is a leading public health problem worldwide. The global estimated prevalence of CKD is 9.1% (range, 8.5–9.8%); the corresponding rate for dialysis was 0·041% (range, 0·037%–0·044%) in 2017 [1]. Cardiovascular disease (CVD) is the primary cause of death in dialysis patients [2]. Indeed, the risk of CVD mortality is 10–30 times higher in the dialysis population than the general population and increases up to 500-fold in the 25–34 year age group [3]. In addition to traditional CVD risk factors, increasing evidence suggests that uremic toxins are non-traditional, CKD-specific CVD risk factors [4].

Uremic toxins consist of three types: water-soluble, small-sized molecules; middle-sized molecules; and protein-bound uremic toxins (PBUTs) [5]. Among the PBUTs, indoxyl sulfate (IS) is one of the most extensively studied and has been shown to have a negative impact on the cardiovascular system [6]. IS is derived from the breakdown of tryptophan by colon microbes [7]. The kidneys achieve high clearance of IS by tubular secretion [8]; however, removal of IS by conventional dialysis is particularly problematic because of its high protein binding [9]. Plasma total IS (tIS) levels in maintenance hemodialysis (MHD) patients are as high as (2.9 ± 1.1) mg/dl, which is 30 times higher than patients with normal renal function [10]. Several potential biological mechanisms have been proposed to underlie IS-induced CVD, including endothelial injury, smooth muscle cell proliferation, atherosclerosis, vascular calcification, cardiomyocyte hypertrophy, and fibrosis [11–15]. These in vitro and animal studies have shown that IS may have a significant role in CVD and the higher mortality rate observed in MHD patients.

However, epidemiologic evidence in support of the association between IS and CVD in MHD patients has been inconsistent. Specifically, in an analysis from the Japan Dialysis Outcomes and Practice Patterns Study, the serum tIS level was significantly associated with all-cause mortality, but the association with cardiovascular events did not reach statistical significance [16]. There was no significant association between the serum tIS level and CVD or all-cause mortality in the Hemodialysis (HEMO) study, but IS was associated with increased CVD mortality in a subgroup analysis in patients with a serum albumin level < 3.6 g/dl [17]. These inconsistencies may be attributed to different patient characteristics and measurement methods. Thus far, there have been no long-term studies investigating the effect of IS on clinical outcomes among Chinese undergoing MHD. Therefore, we conducted this prospective cohort study to determine the association between serum tIS levels, and CVD mortality and all-cause mortality in a large hemodialysis center.

## Materials and methods

### Study population

**Fig. 1:**
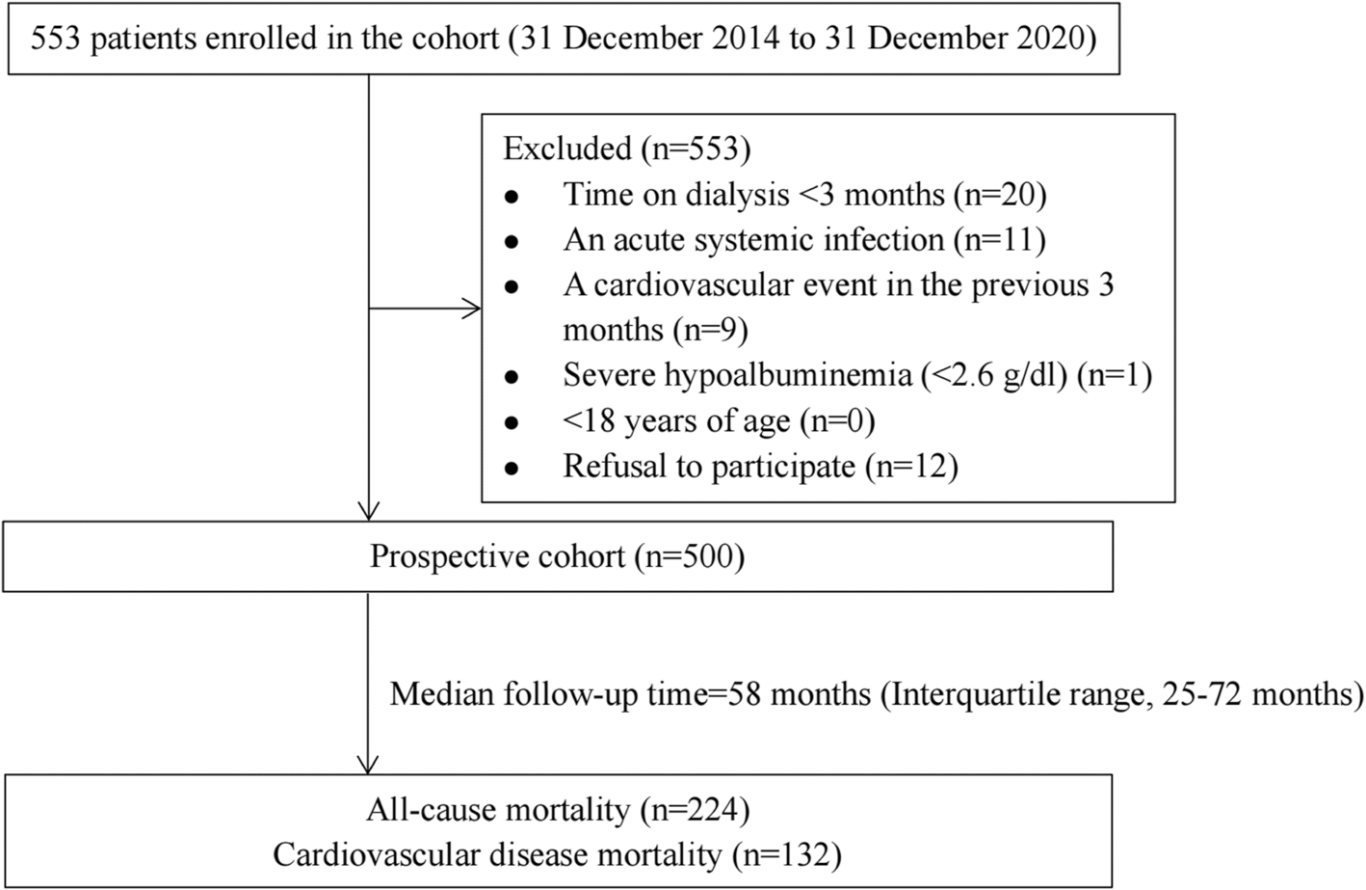
Flow chart indicates patient enrollment and study design

Patients who had received hemodialysis treatment for at least 3 months were enrolled from the Blood Purification Center of Dalian Municipal Central Hospital. Patients with any of the following were not eligible for the study: an acute systemic infection; a cardiovascular event in the previous 3 months, including coronary artery disease, myocardial ischemia, cerebrovascular disease and peripheral artery disease; a malignancy; severe hypoalbuminemia (< 2.6 g/dl); < 18 years of age; or declined participation (Fig. 1). Patients were treated 3 times per week (4 hours per session) with a standard bicarbonate dialysate. The blood flow was 200–300 ml/min and the dialysate flow was 500 ml/min. The enrollment for the prospective HD patient cohort was begun in December 2014. The duration of follow-up was 6 years and ended on 31 December 2020. The study was approved by the Ethical Committee of Dalian Municipal Central Hospital. All participants provided written informed consent.

### Laboratory measurements

Blood was sampled immediately using 5 ml separating gel accelerator tube and 5 ml EDTA anticoagulation tube before the midweek dialysis treatment by the slow flow/stop pump technique. Blood samples were processed (centrifugal, 3500 rpm, 5 minute) within 30 minutes of sampling to obtain plasma, while serum was allowed to clot for 30 minutes at room temperature prior centrifugation (3500 rpm, 5 minute) and stored at − 80 °C until used in assays. Hemoglobin (Hb) was measured using sodium dodecyl lauryl sulfate. Blood urea nitrogen (BUN), creatinine (Cr), albumin (Alb), alanine aminotransferase (ALT), alkaline phosphatase (ALP), potassium (K), sodium (Na), calcium (Ca), phosphorus (P), and chlorine (Cl) were assayed in an on-site biochemistry laboratory using standard autoanalyzer techniques (Siemens, Tarrytown, New York, Germany). The adequacy of dialysis was calculated by measuring urea clearance (Kt/V) using the standard method [18], as follows: Kt/V = −ln(R-0.008 × t) + (4–3.5 × R) × UF/W, where R is the post−/pre-plasma BUN ratio, t is the dialysis session length (in h), UF is the ultrafiltrate volume (in l), and W is the post-dialysis weight (in kg).

Serum tIS was analyzed and determined by high-performance liquid chromatography (HPLC) [19]. Serum samples were deproteinized by the addition of three parts methanol-to-one part serum. The analyses were performed using an Agilent Technologies 1200 Series HPLC (Agilent, US). Serum tIS was detected at 280 nm and appeared at 5.79 min. The limits of detection of this assay were 0.132 mg/L for tIS. Calibration curves were constructed by plotting the peak areas versus the concentrations of analyte with average R^2^ values of 0.999 ± 0.001. Intra-day and inter-day coefficients of variation were 0.06 and 0.07 for tIS.

### Outcome evaluation

During the follow-up period, the primary outcome for our analysis was all-cause mortality. The secondary outcome was cardiovascular mortality. We adjudicated mortality using information recorded on TSS version 2.0 (Therapy Support Suite, Baden Humboldt, German) and hospital records on BS-EAP (version 5.5; B-soft Enterprise Application Portal, HangZhou, China). Cardiovascular mortality included deaths due to coronary events, sudden cardiac death, heart failure, myocardial ischemia, arrhythmias, and cerebrovascular accidents. Causes for death were reviewed by one independent physician, who was blinded to the tIS levels. For each participant, the time-to-event was calculated as the time from the date of entry into the study until the date of the first studied event (mortality), the date of disenrolling from the study, the date of kidney transplantation, or the study completion date, whichever came first.

### Statistical analysis

The normality of all continuous variables was evaluated using the Shapiro-Wilk statistic. The results of continuous variables are expressed as the mean ± standard deviation (SD) or median [quartile1- quartile3], and intergroup comparisons were analyzed using t-tests for normally distributed data or the Mann-Whitney U tests for non-normally distributed data. Categorical variables are expressed as the count with percentage, and differences between the two groups were examined using chi-square tests.

Optimal cut-off points for tIS were determined using X-tile software (version 3.6.1; Yale University School of Medicine, New Haven, CT, USA) [20]. Specifically, the tIS cut-off point was derived from the minimum *P* values from log-rank 2 statistics for the categorical tIS concentration in terms of survival [21]. Survival curves were generated using the Kaplan-Meier method, and differences between the curves were analyzed using the log-rank test. Colinearity among predictors was examined using variance inflation factors.

Univariate and multivariate Cox proportional hazard regression models were performed to calculate hazards ratios (HRs) and the corresponding 95% confidence intervals (CIs) based on the optimal cut-off point of tIS and tIS as continuous variable for all-cause and CVD mortality. We used the Schoenfeld residual test to verify the assumption of proportional hazards in the Cox analysis, and no violations were found (all *P* > 0.05). Statistically significant covariates in the univariate model were included in the multivariate model (*P* < 0.05) with a enter conditional method of analysis, including age, diabetic nephropathy, glomerulus nephritis, hypertension benign renal arteriosclerosis, albumin, urea nitrogen, creatinine, sodium, phosphorus, and chlorine. Restricted cubic spline regression [22] with five knots at the 5th, 35th, 50th, 65th, and 95th centiles were used to estimate the dose-response association between tIS and mortality. Statistical significance was set at a *P* < 0.05 and based on a two-sided test. All analyses were carried out using SAS (version 9.4; SAS Institute, Inc., Cary, NC, USA).

## Results

The baseline characteristics are presented in Table 1. The study consisted of 500 patients on MHD, 53.6% of whom were men. The median age of the participants was 58 years (interquartile range [IQR], 47–68 years) and the median dialysis time was 49 months (IQR, 24–74 months). The median duration of follow-up was 58 months (IQR, 25–72 months). During the follow-up period, all-cause mortality occurred in 224 (44.8%) patients, of which 132 (26.4%) had a cardiovascular cause. The prevalence of diabetic nephropathy, glomerulonephritis, hypertensive nephropathy, polycystic kidney disease, chronic interstitial nephritis, and “other disorders” were 26.8, 42.0, 20.2, 6.2, 2.2, and 2.6%, respectively. The patients were categorized into two groups: low-tIS (< 44.16 μg/ml); and high-tIS (≥44.16 μg/ml). The BUN and P levels were significantly higher in the high-tIS group compared to the low-tIS group (*P* < 0.05). There were no significant differences between the two groups regarding the other variables.

**Table 1:** Baseline characteristics of the study patients according to the optimal cutoff point of total indoxyl sulfate (tIS)

Characteristic	Study population(***n*** = 500)	tIS (ug/ml)		
		< 44.16(***n*** = 448)	≥44.16(***n*** = 52)	***P***
Age, years	58(47–68)	58(47–68)	61(51–71)	0.11
Male, n (%)	268(53.6)	238(53.1)	30(57.7)	0.53
Time on dialysis, months	49(24–74)	49(24–73)	50(24–85)	0.74
Cause of end–stage renal disease, n (%)				0.84
Diabetic nephropathy	134(26.8)	117(26.1)	17(32.7)	0.31
Glomerulonephritis	210(42.0)	191(42.6)	19(36.5)	0.40
Hypertensive benign renal arteriosclerosis	101(20.2)	89(19.9)	12(23.1)	0.59
Polycystic kidney	31(6.2)	29(6.5)	2(3.9)	0.46
Chronic interstitial nephritis	11(2.2)	10(2.2)	1(1.9)	0.89
Other	13(2.6)	12(2.7)	1(1.9)	0.75
Hemoglobin, g/l	109(100–117)	109.5(100–117)	107(98–118)	0.66
Alanine aminotransferase,u/L	11(8–15)	11(8–15)	10(8–16)	0.82
Albumin, g/l	41(39–42)	40.6(39.0–42.2)	40.8(39.5–42.3)	0.57
Alkaline phosphatase, u/l	84(68–114.5)	82.5(68.0–112.5)	88(73–117.5)	0.20
Urea nitrogen, mmol/L	25.7(21.7–29)	25.4(21.4–28.8)	27.6(24.8–31.7)	< 0.01
Creatinine, umol/L	928.5(752.5–1096)	925.0(750.0–1092.5)	960.5(791–1111)	0.24
Kt/V	1.36(1.21–1.53)	1.36(1.21–1.53)	1.30(1.23–1.49)	0.54
Platelet, (×10^9^/L)	191(156.5–228)	193.50(157–229)	184.5(150.5–222)	0.34
Potassium, mmol/L	5.0(4.5–5.6)	5.0(4.4–5.5)	5.3(4.6–5.9)	0.07
Sodium, mmol/l	133.9(131.7–136.4)	133.9(131.8–136.4)	133.9(131.5–136.4)	0.55
Calcium, mmol/l	2.36 ± 0.17	2.36 ± 0.17	2.34 ± 0.18	0.46
Phosphorus, mmol/l	2.0(1.6–2.4)	2.0(1.6–2.4)	2.2(1.8–2.6)	0.04
Chlorine, mmol/l	97.08 ± 4.12	97.15 ± 4.16	96.48 ± 3.73	0.27

Data are displayed as mean ± standard deviation or median (quartile1- quartile3) for continuous variables and number (percent) for categorical variables

*P* values were determined with Student’s t test or Mann-Whitney U tests for continuous variables and chi-square test for categorical variables

All statistical tests are two sided

**Fig. 2:**
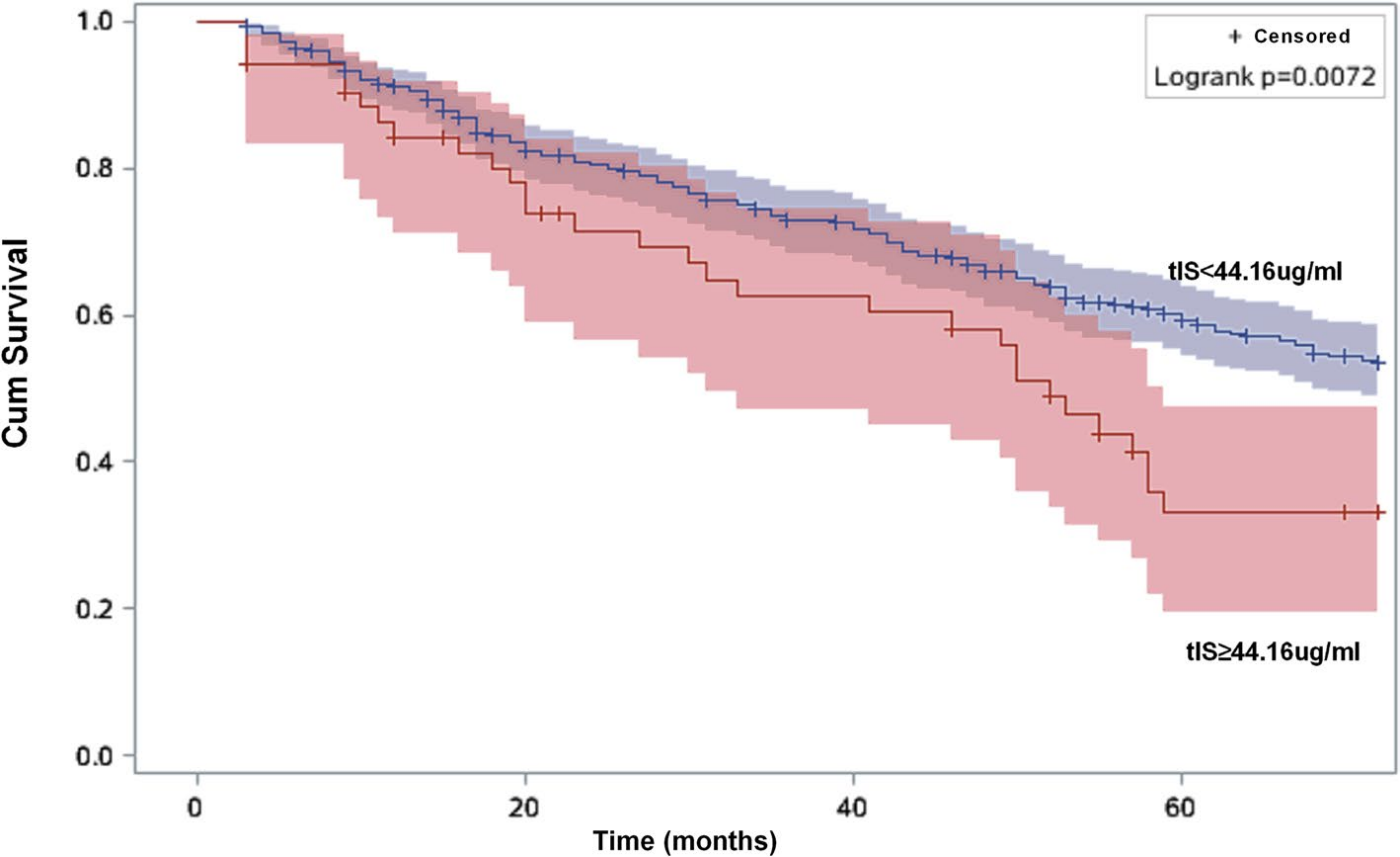
Kaplan-Meier survival estimates of all-cause mortality between the two total indoxyl sulfate subgroups divided by the optimal cutoff value generated by the X-tile program

**Fig. 3:**
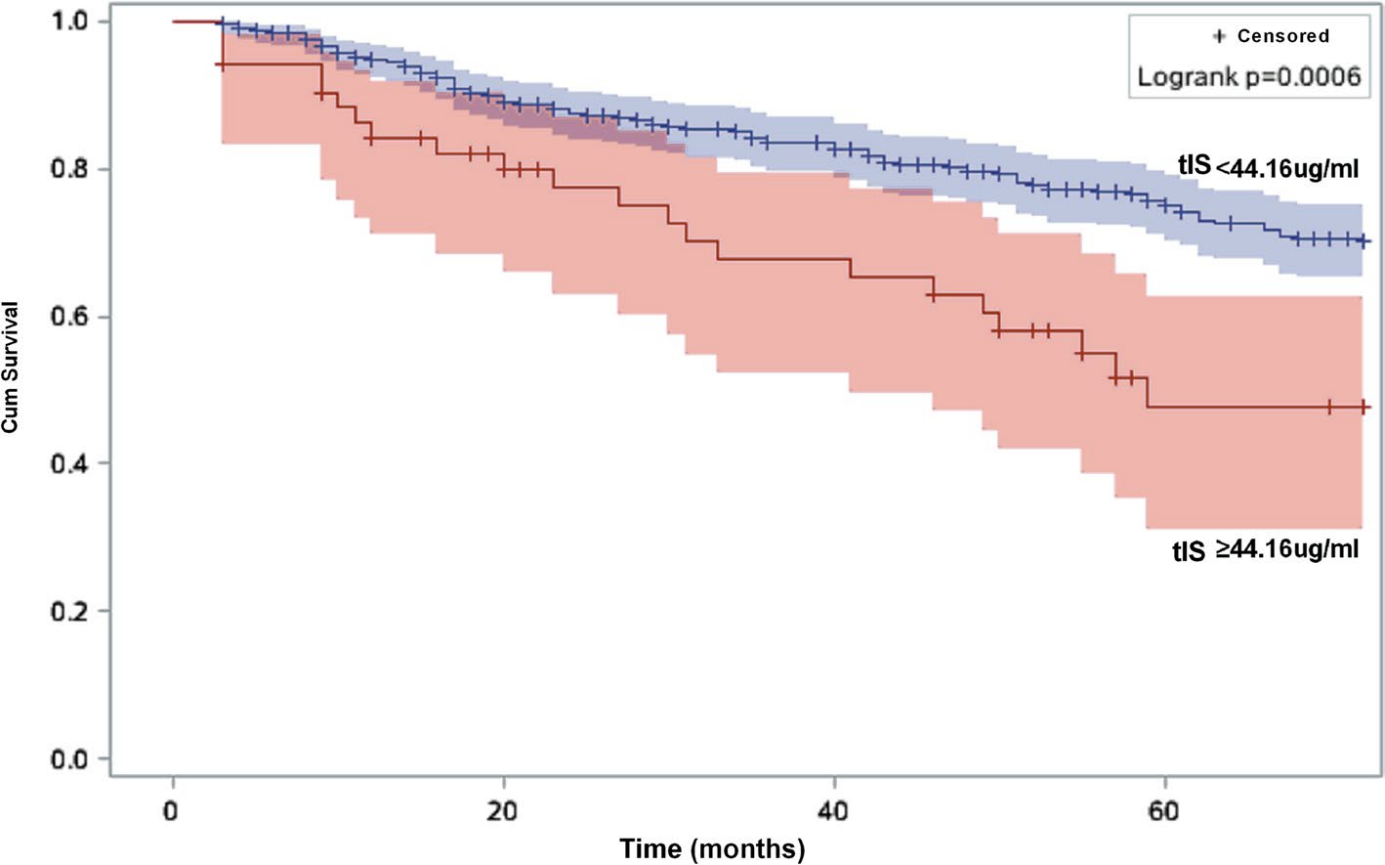
Kaplan-Meier survival estimates of cardiovascular mortality between the two total indoxyl sulfate subgroups divided by the optimal cutoff value generated by the X-tile program

Tables 2 and 3 show the risk estimates of all-cause and CVD mortality. Age, diabetic nephropathy, glomerulonephritis, hypertensive nephropathy, and the Alb, Cr, Na, and Cl levels were significantly associated with all-cause and CVD mortality in the high-tIS group. Figures 2 and 3 show the Kaplan-Meier survival curves of patients with different levels of tIS. A greater number of patients had all-cause and CVD mortality in the high-tIS group compared to the low-tIS group (log-rank, *P* = 0.0072 and 0.006, respectively).

**Table 2:** Univariate Cox regression analysis of prognostic factors for all–cause mortality

Characteristic	HR (95% CI)	***p***
Total Indoxyl Sulfate(≥44.16 vs < 44.16 μg/ml)	1.68 (1.14–2.47)	< 0.01
Total Indoxyl Sulfate (Continuous)	1.02 (1.01–1.03)	< 0.01
Age, years	1.06 (1.05–1.07)	0.01
Gender (Female vs Male)	1.12(0.86–1.46)	0.39
Time on dialysis, months	1.001(0.997–1.004)	0.74
Diabetic nephropathy	2.73(2.09–3.56)	< 0.01
Glomerulonephritis	0.30(0.22–0.42)	< 0.01
Hypertensive benign renal arteriosclerosis	1.45(1.07–1.96)	0.02
Polycystic kidney	0.98(0.58–1.65)	0.94
Chronic interstitial nephritis	0.38(0.10–1.53)	0.17
Hemoglobin, g/l	0.99 (0.98–1.00)	0.01
Alanine aminotransferase,u/L	1.000 (0.999–1.001)	0.36
Albumin, g/l	0.82(0.77–0.86)	< 0.01
Alkaline phosphatase, u/l	1.001(1.000–1.002)	0.20
Urea nitrogen, mmol/L	0.97(0.94–0.99)	0.01
Creatinine, umol/L	0.998(0.997–0.998)	< 0.01
Kt/V	0.64(0.37–1.12)	0.12
Platelet, (×10^9^/L)	1.00(0.998–1.003)	0.70
Potassium, mmol/L	0.91(0.77–1.07)	0.24
Sodium, mmol/l	0.92(0.89–0.96)	< 0.01
Calcium, mmol/l	0.83(0.38–1.77)	0.62
Phosphorus, mmol/l	0.77(0.61–0.98)	0.04
Chlorine, mmol/l	0.96(0.93–0.99)	0.01

*Abbreviation*: *CI* confidence interval, *HR* hazards ratio

**Table 3:** Univariate Cox regression analysis of prognostic factors for cardiovascular mortality

Characteristic	HR (95% CI)	***P***
Total Indoxyl Sulfate(≥44.16 vs < 44.16 μg/ml)	2.18(1.38–3.46)	< 0.01
Total Indoxyl Sulfate (Continuous)	1.02(1.01–1.03)	< 0.01
Age, years	1.05(1.04–1.07)	0.01
Gender (Female vs Male)	1.04(0.74–1.46)	0.84
Time on dialysis, months	1.002(0.998–1.006)	0.33
Diabetic nephropathy	2.99(2.12–4.22)	< 0.01
Glomerulonephritis	0.28(0.18–0.43)	< 0.01
Hypertensive benign renal arteriosclerosis	1.56(1.06–2.29)	0.02
Polycystic kidney	0.88(0.43–1.81)	0.73
Hemoglobin, g/l	0.997(0.986–1.008)	0.58
Alanine aminotransferase,u/L	1.001(1.000–1.002)	0.12
Albumin, g/l	0.84(0.78–0.89)	< 0.01
Alkaline phosphatase, u/l	1.001(1.000–1.002)	0.14
Urea nitrogen, mmol/L	0.99(0.96–1.02)	0.46
Creatinine, umol/L	0.998(0.998–0.999)	< 0.01
Kt/V	0.46(0.22–0.95)	0.04
Platelet, (×10^9^/L)	1.001(0.998–1.004)	0.48
Potassium, mmol/L	1.04(0.84–1.29)	0.73
Sodium, mmol/l	0.92(0.87–0.96)	< 0.01
Calcium, mmol/l	0.69(0.26–1.86)	0.46
Phosphorus, mmol/l	0.96(0.71–1.30)	0.79
Chlorine, mmol/l	0.95(0.91–0.99)	0.02

All statistical tests are two sided

*Abbreviation*: *CI* confidence interval, *HR* hazards ratio

**Fig. 4:**
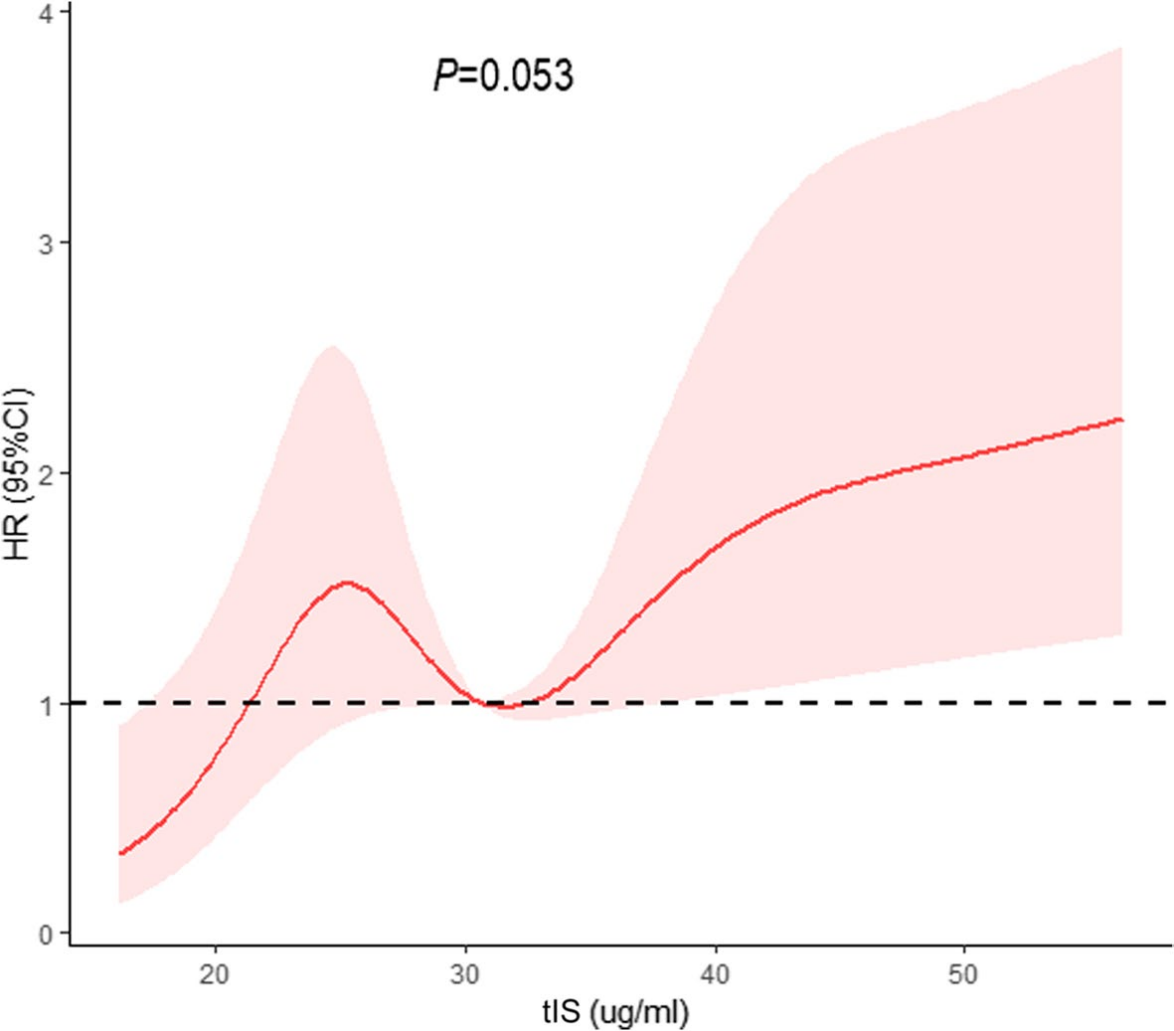
The dose-response relationship of total indoxyl sulfate with the risk of cardiovascular disease mortality, estimated by restricted cubic spline models. The red solid line and the shaded area represent the estimated HRs and their 95% CIs, respectively

**Fig. 5:**
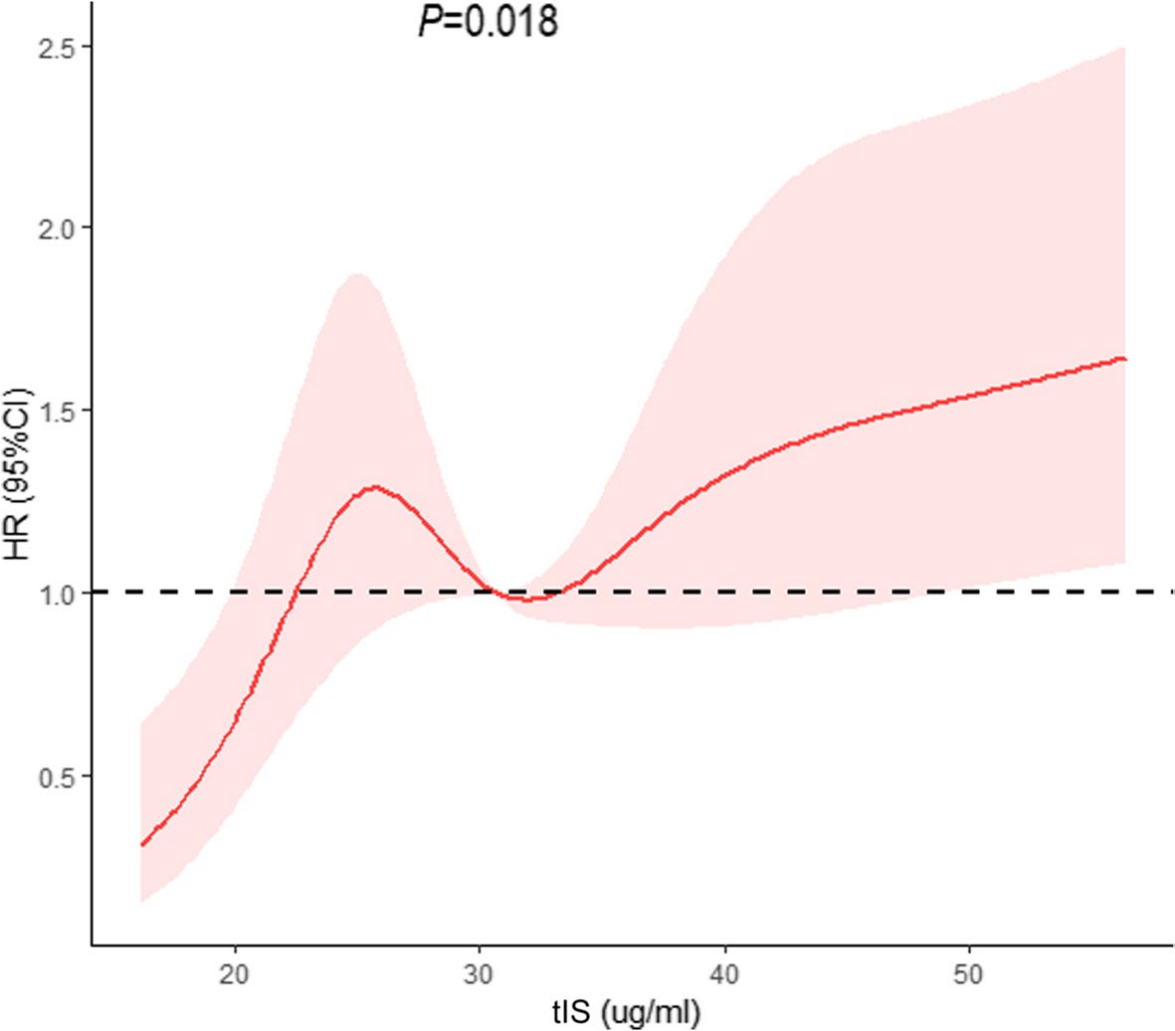
The dose-response relationship of total indoxyl sulfate with the risk of all-cause mortality, estimated by restricted cubic spline models. The red solid line and the shaded area represent the estimated HRs and their 95% CIs, respectively

A multivariate Cox proportional hazards model was constructed to compare the association between the level of tIS and all-cause and CVD mortality. In the fully adjusted model, MHD patients with high tIS concentrations had an increased risk (HR = 1.02, 95% CI = 1.01–1.03) of all-cause mortality when tIS was entered as a continuous variable. In addition, age, diabetic nephropathy, and the Alb level were shown to be independently associated with all-cause mortality (*P* < 0.05; Table 4). MHD patients with high tIS concentrations had an increased risk of CVD mortality when tIS was entered as a dichotomous (HR = 1.76, 95% CI = 1.10–2.82) or continuous variable (HR = 1.02, 95% CI = 1.01–1.03). Additionally, age, diabetic nephropathy, the Alb level, and Kt/V were independently associated with CVD mortality (*P* < 0.05; Table 5). In the dose-response analysis, there was a linear dose-response relationship between tIS and risk of CVD mortality (*P*_nonlinear_ > 0.05) (Fig. 4), while a nonlinear association (*P*_nonlinear_ < 0.05) between tIS and risk of all-cause mortality (Fig. 5) after adjusting aforementioned covariates. In addition, the finding that the variance inflation values were < 5 in the linear regression model is considered to indicate the absence of colinearity among the predictors (data not shown).

**Table 4:** Multivariable Cox regression analysis of prognostic factors for all-cause mortality

Characteristic	HR (95% CI)	***P***	HR (95% CI)	***P***
Total Indoxyl Sulfate(≥44.16 vs < 44.16 μg/ml)	1.29(0.87–1.92)	0.21	–	–
Total Indoxyl Sulfate (Continuous)	–	–	1.02(1.01–1.03)	< 0.01
Age, years	1.05(1.04–1.06)	< 0.01	1.05(1.04–1.06)	< 0.01
Diabetic nephropathy	2.00(1.25–3.16)	< 0.01	1.96(1.24–3.11)	< 0.01
Glomerulonephritis	0.77(0.47–1.27)	0.31	0.77(0.47–1.26)	0.29
Hypertensive benign renal arteriosclerosis	1.29(0.80–2.10)	0.30	1.26(0.78–2.04)	0.35
Hemoglobin, g/l	0.99(0.98–1.00)	0.20	0.99(0.98–1.00)	0.13
Albumin, g/l	0.91(0.86–0.96)	< 0.01	0.91(0.86–0.97)	< 0.01
Urea nitrogen, mmol/L	1.00(0.97–1.03)	0.86	0.99(0.96–1.02)	0.52
Creatinine, umol/L	1.00(0.999–1.001)	0.50	1.00(0.999–1.00)	0.40
Sodium, mmol/l	1.00(0.94–1.05)	0.86	0.99(0.94–1.05)	0.73
Phosphorus, mmol/l	1.08(0.80–1.46)	0.60	1.11(0.82–1.50)	0.49
Chlorine, mmol/l	0.97(0.92–1.01)	0.16	0.97(0.92–1.02)	0.20

All statistical tests are two sided

*Abbreviation*: *CI* confidence interval, *HR* hazards ratio

**Table 5:** Multivariable Cox regression analysis of prognostic factors for cardiovascular mortality

Characteristic	HR (95% CI)	***P***	HR (95% CI)	***P***
Total Indoxyl Sulfate(≥44.16 vs < 44.16 μg/ml)	1.76(1.10–2.82)	0.02	–	–
Total Indoxyl Sulfate (Continuous)	–	–	1.02(1.01–1.03)	< 0.01
Age, years	1.04(1.02–1.06)	< 0.01	1.04(1.03–1.06)	< 0.01
Diabetic nephropathy	2.68(1.39–5.19)	< 0.01	2.58(1.33–4.99)	< 0.01
Glomerulonephritis	0.97(0.48–2.00)	0.94	0.93(0.45–1.90)	0.84
Hypertensive benign renal arteriosclerosis	1.86(0.94–3.67)	0.07	1.76(0.89–3.47)	0.10
Albumin, g/l	0.90(0.83–0.97)	< 0.01	0.91(0.84–0.98)	0.02
Creatinine, umol/L	1.00(0.999–1.001)	0.84	1.00(0.999–1.001)	0.75
Kt/V	0.40(0.17–0.95)	0.04	0.40(0.17–0.94)	0.04
Sodium, mmol/l	0.99(0.93–1.07)	0.88	0.98(0.92–1.06)	0.65
Chlorine, mmol/l	0.96(0.90–1.02)	0.17	0.97(0.91–1.03)	0.20

All statistical tests are two sided

*Abbreviation*: *CI* confidence interval, *HR* hazards ratio

## Discussion

In the current study, we evaluated the role of tIS and other important clinical variables in all-cause and CVD mortality among MHD patients. We showed that elevated tIS concentrations were associated with increased risks for all-cause and CVD mortality, which indicated that tIS is a predictor for all-cause and CVD mortality in MHD patients. Of particular importance, there is a novel finding that a linear dose-response pattern was found for the association between tIS and risk of CVD mortality.

Serum tIS levels were significantly related to all-cause and CV mortality, and this finding is similar to the findings reported in previous studies. Specifically, a meta- analysis including 11 studies conducted by Lin et al. demonstrated that elevated levels of IS were associated with an increased mortality in patients with CKD, but in contrast, IS was not associated with an increased risk of cardiovascular mortality events [23]. Besides, a cohort study involving 1170 hemodialysis (HD) patients in Japan [16] revealed a positive relationship between tIS levels and all-cause mortality. Another prospective cohort study involving 258 HD patients in China also showed that a high plasma IS level was associated with a higher risk of the first heart failure event [24]. In a cohort involving 139 patients in France with different stages of CKD (stage 2–5 on dialysis), baseline tIS had a positive relationship with all-cause and CVD mortality [25]. More importantly, the crude event rates for all-cause and CVD mortality in our study was similar with abovementioned studies. In contrast, other studies [17, 26] did not show a significant association between IS and all-cause or CVD mortality. Lin et al. [21] reported that serum tIS was not associated with all-cause mortality or major adverse cardiovascular events in a prospective cohort study of 200 HD patients in Taiwan. Lin et al. [21] used median cut-off points for the serum tIS, while we used X-tile software, which demonstrated population cut-off points based on marker expression and provides superior assessment of biological relationships between IS and outcomes [20]. Additionally, in a *post-hoc* analysis of 1273 HD patients in the HEMO trial [17], there was a lack of association between tIS and all-cause or CVD mortality after a mean follow-up duration of 2.3 years. HD patients are known to have fewer outcomes with a shorter follow-up time, and thus our ability to estimate this association between IS and outcomes with high precision may have been limited. In addition, tIS was measured by stable isotope dilution liquid chromatography/mass spectrometry/mass spectrometry (LC-MS/MS) [27], while we used HPLC, which might also be the reason for the discrepancy with our results.

There is increasing evidence that shows IS contributes to the mechanism underlying cardiovascular events., IS promotes the expression of myocardial hypertrophic protein and stimulates cardiac fibroblast collagen synthesis by activating the NLRP3 inflammasome signaling pathway [28], and p38 mitogen-activated protein kinase, p42/44 mitogen-activate protein kinase, and NF-kB pathways [29], thereby aggravating myocardial fibrosis and hypertrophy with enhanced oxidative stress and reduced antioxidant capacity. Second, IS promotes proliferation of vascular smooth muscle cells by activating the MAPK pathway and upregulating expression of osteoblast-specific proteins, which can induce aortic wall thickening and aortic calcification [12–14]. Furthermore, IS causes endothelial cell damage and decreases new blood vessel formation by inducing the expression of NADPH oxidase to reduce the production of NO [30] and activating the p53 and NF-κB pathways to promote senescence of endothelial progenitor cells [31]. Therefore, IS may be a potential predictor of cardiovascular events in end-stage renal disease patients.

Our study had several strengths. First, the sample size was adequate to explore the associations between tIS levels, and all-cause and CVD mortality in MHD patients. Second, the outcomes, including all-cause and CVD mortality in MHD patients, were prospectively observed over a relatively long duration of follow-up (72 months). Third, we used the method described by Camp et al. [17] to determine the optimal cut-off point for the serum tIS concentration, which avoided blinding, as was done in previous studies [16, 24]. There were some limitations in our study. First, serum tIS levels were measured at a single time point, although the concentration may change over time. Single-point measurements may not reflect substantial intra-individual variability over time and may increase the probability of random measurement error; however, the findings can provide some clues for corollary studies. Second, we used total concentrations of IS as the outcome predictor rather than free concentrations. Free IS concentrations are presumed to be a better indicator for potential toxicity when tissues are exposed to free solutes. The free solute levels are also more likely to be influenced by other unmeasured protein-bound uremic toxins that may replace IS from their binding sites, leading to higher free IS levels [17, 32]. Therefore, the tIS concentration selected for this study may be more representative. Third, due to the limited data, this study may have been influenced by some unmeasured confounders, such as residual renal function (RRF) or proxy of RRF such as the use of diuretics, hemodialysis pattern, smoking, alcohol consumption, and body mass index. Also, as with all observational studies, we cannot exclude the possibility of residual confounding. Fourth, patients enrolled in this study were from a single dialysis center, thus their representability was weak. As a result, the findings may not be generalizable to the overall hemodialysis population, and further verification is needed with additional studies in the future.

In conclusion, the novel finding in our study was that high concentrations of tIS may be associated with an increased risk of all-cause and CVD mortality among MHD patients. Greater effort, particularly detailed prevention strategies for reducing tIS levels, should be performed to decrease the associated mortality in the future.

## Data Availability

The datasets used and/or analyzed during the current study are available from the corresponding author upon reasonable request.

## Abbreviations

ALP: Alkaline phosphatase
ALT: Alanine aminotransferase
Alb: Albumin
BUN: Blood urea nitrogen
Cl: Chlorine
Ca: Calcium
Cr: Creatinine
CKD: Chronic kidney disease
CIs: Confidence intervals
CVD: Cardiovascular disease
Hb: Hemoglobin
HEMO: Hemodialysis
HRs: Hazard ratios
HPLC: High-performance liquid chromatography
HD: Hemodialysis
IS: Indoxyl sulfate
IQR: Interquartile range
K: Potassium
Kt/V: Urea clearance
LC-MS/MS: Liquid chromatography/mass spectrometry/mass spectrometry
MHD: Maintenance hemodialysis
Na: Sodium
PBUTs: Protein-bound uremic toxins
P: Phosphorus
RRF: Residual renal function
SD: Standard deviation
tIS: Total indoxyl sulfate
